# *Lactobacillus plantarum* KLDS1.0318 Ameliorates Impaired Intestinal Immunity and Metabolic Disorders in Cyclophosphamide-Treated Mice

**DOI:** 10.3389/fmicb.2019.00731

**Published:** 2019-04-12

**Authors:** Yueyue Meng, Jing Wang, Zhiyu Wang, Guofang Zhang, Libo Liu, Guicheng Huo, Chun Li

**Affiliations:** Key Laboratory of Dairy Science, Ministry of Education, College of Food Science, Northeast Agricultural University, Harbin, China

**Keywords:** *Lactobacillus plantarum* KLDS1.0318, cyclophosphamide, intestinal mucosal immunity, cytokine, intestinal metabolism

## Abstract

Cyclophosphamide (CTX), a clinically important antineoplastic drug, also leads to some side effects such as nausea, vomiting and diarrhea in the consumer. In this study, *Lactobacillus plantarum* (*L. plantarum*) KLDS1.0318 preserved in our laboratory was orally administered to CTX-treated mice to explore its potential effects to attenuate the toxic effects of CTX-induced by modulating intestinal immune response, promoting intestinal integrity and improving metabolic profile. BALB/c mice were randomly divided into six groups including normal control group (NC; non-CTX with sterile saline), model control group (MC; CTX-treated with sterile saline), CTX-treated with *L. plantarum* KLDS1.0318 (10 mL/kg) groups with three different doses (KLDS1.0318-L, 5 × 10^7^ CFU/mL; KLDS1.0318-M, 5 × 10^8^ CFU/mL; KLDS1.0318-H, 5 × 10^9^ CFU/mL), and CTX-treated with levamisole hydrochloride (40 mg/kg) as a positive control (PC) group. After receiving the bacterium for 20 days, samples of small intestine and colonic contents were collected for different analyses. The results revealed that the levels of cytokines secreted by Th1 cells (IL-2, IFN-γ, and TNF-α) and Th2 cells (IL-4, IL-6, and IL-10) in probiotic treatment groups were significantly higher than those in the MC group. Histopathological results showed that *L. plantarum* KLDS1.0318 favorably recovered CTX-induced abnormal intestinal morphology by improving the villus height and crypt depth as well as quantity of goblet cells and mucins production. Compared to CTX alone-treated group, the production of short-chain fatty acids (SCFAs) were significantly increased and the levels of pH and ammonia were decreased significantly with high dose *L. plantarum* KLDS1.0318 supplementation. Compared with mice in CTX alone-treated group, mice in three groups of KLDS1.0318 had increased *Bifidobacterium* and *Lactobacillus* and decreased *Escherichia* and *Enterococcus* in their cecal content. The present findings suggested that *L. plantarum* KLDS1.0318 could be of significant advantage to mitigate the harmful effects of CTX and improve the intestinal health in mice.

## Introduction

Cyclophosphamide (CTX) belongs to the oxazaphosphorine family of mustard-alkylating agents. It is one of the most successful antineoplastic agents synthesized in 1958 by Norbert Brock (Arnold et al., 1958; Madondo et al., 2016). Even today, owning to its direct cytotoxic effect on cancer cells, CTX is still remains one of the few chemotherapeutic drugs used to treat a range of cancers including lymphomas and solid tumors (breast cancer, ovarian cancer etc.) (Baumann and Preiss, 2001). Moreover, CTX is also an effective immunosuppressive agent and widely used in blood and marrow transplantation (BMT) and for the treatment of patients with a variety of autoimmune disorders (Perini et al., 2007; Uber et al., 2010). Nevertheless, CTX’s unique metabolism and inactivation by aldehyde dehydrogenase are responsible for its distinct cytotoxic properties (Emadi et al., 2009). Therefore, despite its medical effectiveness, the activated metabolites produced by CTX can interfere with DNA replication and damage mitochondrial membranes as well as lysosomal membrane, which causes damage to both tumor tissue and normal tissue (Al-Nasser, 1998; Sudharsan et al., 2006). The toxicity of CTX can generate deleterious side effects in human health and experimental animals, with dosage and duration of therapy being the principal risk factors (Bhattacharya et al., 2003; Habibi et al., 2015; Bhatt et al., 2017). Nausea and vomiting are common side effects of CTX administration and are especially obvious with intermediate and high dosages. Furthermore, diarrhea is uncommon with oral CTX administration, but may occur following high-dose treatment (Emadi et al., 2009). Chemotherapy treatment increases intestinal epithelial cell apoptosis and impacts the gut microbiota profile, causing acute gastrointestinal mucosal damage and metabolic disorders that probably account for the occurrence of the symptoms (Daniele et al., 2001; Zuo et al., 2014). For recovering compromised epithelial barrier and reducing the side effects of chemotherapy drugs, new therapeutic options including probiotics and peptides are evolving (Baumgart and Dignass, 2002; Prisciandaro et al., 2011).

Probiotics are defined as live microorganisms which are able to confer health benefits on the host when administered in adequate amounts (Patel et al., 2015). They are well known to alter the gut microbiota profile, act as the competitive inhibition with other adverse bacterial components via adhesion to the mucosa and epithelium, reinforcing the intestinal epithelial barrier function and modification of the intestinal immune responses in favor of the host (Thomas and Versalovic, 2010; Bermudezbrito et al., 2012). A case study discussed previously have indicated that a multispecies combination of probiotics successfully treated patients with chemotherapy-induced diarrhea via recovering damaged intestinal lining (Abd et al., 2009). In addition, prior study has shown that Nanometric *Lactobacillus plantarum* (*L. plantarum*) nF1 could be used to recover normal immunity in mice immunosuppressed by CTX treatment (Choi et al., 2017). Prior study has also shown that dietary supplementation with *L. plantarum* B1 could increase the counts of lactic acid bacteria and concentrations of SCFAs in the intestine (Peng et al., 2016).

*L. plantarum* KLDS1.0318, one kind of probiotic bacteria, was freshly identified and preserved in our laboratory. *L. plantarum* KLDS1.0318 has been previously investigated for its ability to recover immunity of the immunocompromised mice (Meng et al., 2018). However, the effects of *L. plantarum* KLDS1.0318 on intestinal immune function and its action mechanism are not clear. Accordingly, the objective of this study was to explore intestinal immunity improving capability of *L. plantarum* KLDS1.0318 intervention and whether such treatment would lead to restored impaired intestinal function in CTX-treated mice.

## Materials and Methods

### Ethics Statement

All the experiments were carried out according to animal ethics guidelines and approved protocols of the Animal Care and Use Committee of Northeast Agricultural University (SRM-06).

### Chemicals

Cyclophosphamide (CTX) was purchased from Beijing solarbio science and technology Co., Ltd. (Beijing, China). Levamisole hydrochloride was purchased from Sigma Co. (St. Louis, MO, United States). Enzyme-Linked Immunosorbent Assay (ELISA)-based cytokine kits were purchased from BOSTER Biological Technology Co., Ltd. (Wuhan, China). All other reagents used were of analytical grade and purchased from Tianjin Kemiou Chemicals and Reagents Co., Ltd. (Tianjin, China).

### Preparation of Bacterial Strain

KLDS1.0318 (preserved at Key Laboratory of Dairy Science, Ministry of Education, Northeast Agricultural University) was grown by inoculating (2% v/v) in Man-Rogosa-Sharpe (MRS) broth (peptone 10.0 g, beef extract 10.0 g, glucose 20.0 g, yeast extract powder 5.0 g, sodium acetate 5.0 g, di-potassium hydrogen phosphate 2.0 g, tri-ammonium citrate 2.0 g, magnesium sulfate 0.5 g, manganese sulfate 0.05 g, tween-80 1.0 g, distilled water 1000 mL, pH 6.5, autoclaved for 15 min at 121°C) (Guo et al., 2015) followed by incubation for 18 h at 37°C. In the pre-experiment, we have assessed the approximate concentrations of viable bacterium by the plate count method. The concentrations of KLDS1.0318 were found to reach 5 × 10^9^ colony forming units (CFU)/mL when it was cultured under the same condition as described above. For the preparation of gavages, the bacterium were harvested by centrifugation (2000 ×*g*, 10 min, 4°C), washed twice with sterile phosphate-buffered saline (PBS), and removal of the supernatant. Then according to the measured content of viable cells, the bacterial strain was resuspended and diluted in fresh PBS to produce suspensions of designated doses for oral administration.

### 16S rRNA Gene Sequence Analysis

KLDS1.0318 genomic DNA was isolated using the DNeasy Tissue kit (Qiagen, Hilden, Germany) according to the manufacturer’s instructions. The universal primer (27F/1492R) was used for PCR and the amplification conditions were as follows: initial denaturation step at 94°C for 3 min, 35 cycles of denaturation at 94°C for 30 s, annealing at 55°C for 30 s, elongation at 72°C for 1 min and final extension at 72°C for 5 min (Romanovskaya et al., 2004). The PCR product was then sent for sequencing to the Comate Bioscience Co., Ltd (Jilin, China). The sequencing result was compared with closely related sequences available from GenBank with the use of the BLASTN software. Construction of a phylogenetic tree was performed by the neighbor-joining method by using MEGA 5.0 software package (Chun Yan et al., 2012).

### Experimental Animals

Ninety female Specific Pathogen-Free (SPF) BALB/c mice with the body weight of 20.0 ± 2.0 g were purchased from Beijing Vital River Laboratory Animal Technology Co., Ltd. (Beijing, China, certificate number: SCXK2012-0001). Animals were acclimatized to the laboratory condition for 1 week before starting the trial. They were housed in plastic cages with proper bedding material in a room with controlled temperature (23 ± 1°C) and relative humidity (50 ± 10%). The mice were kept on 12 h light and 12 h dark, fed under standard managemental conditions. Animals used in this study were cared for in accordance with the Guidelines for the Care and Use of Laboratory Animals published by the U.S. National Institutes of Health (NIH Publication 85-23, 1996), and all experimental procedures were performed according to the Animal Care and Use Committee, Northeast Agricultural University.

### Experimental Design

After 1 week adaptation period, the mice were randomly divided into six groups as follows: normal control group (NC; non-CTX with sterile saline), model control group (MC; CTX-treated with sterile saline); CTX-treated with *L. plantarum* KLDS1.0318 groups with three different doses (KLDS1.0318-L, 5 × 10^7^ CFU/mL; KLDS1.0318-M, 5 × 10^8^ CFU/mL; KLDS1.0318-H, 5 × 10^9^ CFU/mL), and CTX-treated with levamisole hydrochloride (40 mg/kg) as a positive control (PC) group. Mice in the MC group, the PC group and the three bacterium treatment groups were injected intraperitoneally with CTX 80 mg/kg of body weight in sterile saline once a day for 3 consecutive days to induce intestinal mucosal damage and metabolism disorders, while the mice in the NC group were subjected to intraperitoneal injection of sterile saline as a control. Later mice in the PC group and the three bacterium treatment groups were given oral administration of levamisole hydrochloride or bacterium, whereas the other two groups were given oral administration of sterile saline. All treatments were conducted with 10 mL/kg body weight once daily for 20 days.

### Sample Collection

Mice were sacrificed by cervical dislocation on day 20. The day when mice received the bacterium after treatment with CTX was considered as the first day of the experiment. The small intestine was excised and washed with 0.9% normal saline (NaCl), prepared for further analysis. The colons and ceca were aseptically removed and placed on an ice-cold plate, the cecal contents and colon feces samples (equally divided into 3 portions) were then collected in sterile tubes and stored at -80°C for further analysis.

### Measurement of Cytokines by ELISA

Samples of small intestine were minced to small pieces and homogenized them in ice-cold normal saline (w:v = 1:10) with a glass homogenizer (Shanghai, China) on ice. The resulting suspension was centrifuged for 15 min at 3000 rpm. The supernatant of the homogenates was harvested and stored in aliquot at -20°C for later use. The levels of cytokines interleukin-2 (Mouse IL-2 ELISA Kit; EK0398), interleukin-4 (Mouse IL-4 ELISA Kit; EK0405), interleukin-6 (Mouse IL-6 ELISA Kit; EK0411), interleukin-10 (Mouse IL-10 ELISA Kit; EK0417), tumor necrosis factor-alpha (Mouse TNF Alpha ELISA Kit; EK0527) and interferon-gamma (Mouse IFN Gamma ELISA Kit; EK0375) were determined in the supernatant according to the instructions of the manufacturer. The results were expressed as the concentration of cytokines per milliliter of supernatant from intestinal tissue homogenate by standard cytokines provided in the kits.

### Histopathological Examination

The small intestinal tissues were prepared for histological observation using the method described previously (Muhammad et al., 2017). In brief, samples of jejunum tissue were fixed in 10% neutral formalin for 24 h, after being processed in a series of graded ethanol and dimethyl benzene, then embedded in molten paraffin. Tissue sections of 4 μm thickness were sliced and mounted on glass slides. The slides were then stained with hematoxylin and eosin (HE) after deparaffinization. Then the stained slides were mounted in neutral balsam and covered with coverslips. The histological differences between the groups were examined under a light microscope (Nikon E100, 40× magnification), and the images were acquired by a digital camera.

The intestinal villus length and crypt depth were measured via Image Pro Plus 6.0 software (Media Cybernetics, MD, United States). The length of each villus was measured from the top of the villus to the crypt transition, and the definition of the crypt depth was the invagination between two villi. The heights of 5 villi and the depths of 5 crypts were measured each animal (Xie et al., 2016).

The deparaffinization tissue sections above mentioned were stained with alcian blue periodic acid schiff staining kit (AB-PAS) (Solarbio, Beijing, China) according to the manufacturer’s protocols. The images were acquired by a camera under biological microscopes (200× magnification). The total mucins areas and quantities of goblet cells in epithelial cells of the small intestine tissues were measured and counted using Image pro plus software 6.0, respectively. The results were expressed as the average of mucins area and the number of goblet cells of 5 intestinal villi.

### SCFAs Analysis

The levels of SCFAs were measured as the method described previously (Hu et al., 2012). Briefly, 100.00–200.00 mg of feces was put into a stoppered tube in an iced water bath, diluted with deionized water at a ratio of 1:9 and mixed on a vortex mixer for 2 min. The tube was subjected to ultrasound for 5 min, then kept in the ice-cold water for 10 min and centrifuged at 4800 ×*g* for 20 min at 4°C. The supernatants were analyzed by gas chromatography (GC) for the concentration of SCFAs. An Agilent 6890 N GC system equipped with a flame ionization detector (FID) and an N10149 automatic liquid sampler (Agilent, United States) was used for chromatographic analysis. GC column of 30 m × 0.32 mm (length × inside diameter) coated with 0.50 μm film thickness (HP-INNOWAX, 190901N-213, J and W Scientific, Agilent Technologies Inc., United States) was used. Nitrogen was supplied as the carrier gas at a flow rate of 19.0 mL/min with a split ratio of 1:10. The initial oven temperature was 100°C and was kept there for 30 s and then raised to 180°C by 4°C /min. The temperatures of the FID and injection port were 240°C. The flow rates of hydrogen and air were 30 and 300 mL/min, respectively. 0.2 μL of sample was injected and each of it was run for 20.5 min for GC analysis.

### Measurement of Colon pH

Another portion of the feces samples was diluted using a 9:1 ratio of deionized water to fecal sample (Chung et al., 2007), and then the pH value was determined using a micro-pH meter (Shanghai analytic corp., Shanghai, China).

### Measurement of Ammonia Content

The ammonia concentration of the feces was measured using the method of indophenol blue colorimetric (Lewandowska and Falkowska, 2004). 50.00–100.00 mg samples of feces were added with 500 μL 0.2 g/mL trichloroacetic acid solution and 500 μL 2 mol/L potassium chloride solution, mixed on a vortex mixer for 1 min and then centrifuged at 5000 g for 2 min. The supernatants were filtered and diluted with deionized water at a ratio of 1:19, NH4^+^ developing solution A (0.5 mol/L phenol, 0.8 mmol/L sodium nitroprusside) and NH4^+^ developing solution B (4% sodium hypochlorite solution in 0.5 mol/L sodium hydroxide) were added to the samples of supernatants in tubes. The tubes were then kept at 37°C in a water bath for 30 min to completely blend. The concentration of ammonia was determined using a TU-1900 double-beam UV-vis spectrophotometer with the absorbance at 630 nm.

### Real-Time PCR Assay

Total microbiota genomic DNA from cecal contents was extracted using a QIAamp DNA stool mini kit (Qiagen, Germany). The specific primers were designed using Primer 5.0 and synthesized by Comate Bioscience Co., Ltd. for real-time PCR (Table 1). The PCR reactions were carried out in a total volume of 25 μL containing 1 μL of template DNA, 12.5 μL of SYBR Premix Ex Taq II, 1 μL each of forward and reverse primer and 9.5 μL of ddH_2_O. The amplification conditions were as follows: initial denaturation step at 95°C for 5 min, 30 cycles of 95°C for 10 s (denaturation), 95°C for 15 s (denaturation), 55–70°C for 20 s (annealing), 72°C for 45 s (extension) and 72°C for 5 min (final extension step). The results were expressed as the log of the copy number of the target bacterial DNA per gram of feces (wet weight) (Whelan et al., 2003).

**Table 1:** Primer sequence of qRT-PCR.

Genera	Primer Sequences (5′3′)
*Bifidobacterium*	For: TCGCGTCTGGTGTGAAAG Rev: CCACATCCAGCGTCCAC
*Lactobacillus*	For: AGCAGTAGGGAATCTTCCA Rev: CACCGCTACACATGGAG
*Escherichia*	For: GGAGCAAACAGGATTAGATACCC Rev: AACCCAACATTTCACAACACG
*Enterococcus*	For: CCCTTATTGTTAGTTGCCATCATT Rev: ACTCGTTGTACTTCCCATTGT


*For and Rev indicate forward and reverse, respectively.*

### Statistical Analysis

All the tests were performed in triplicates unless specifically stated otherwise, and the results were expressed as mean ± standard deviation (SD). Significance of the data was analyzed with SPSS 20.0 software (SPSS Inc., Chicago, IL, United States). The statistical significance of data comparisons among the various groups was determined using one-way analysis of variance (ANOVA), followed by Duncan’s multiple range test. Values of *p* < 0.05 were considered statistically significant.

## Results

### Construction of Phylogenetic Tree

**FIGURE 1:**
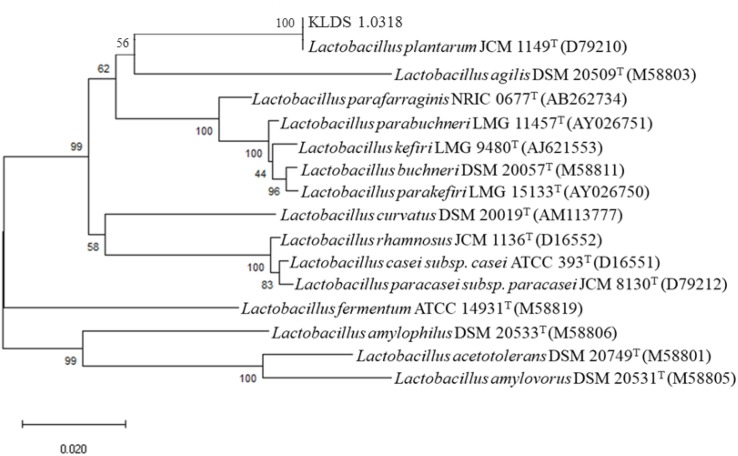
Phylogenetic trees of the strain KLDS1.0318 based on comparative analysis of the 16S rRNA genes sequence. Bootstrap values based on 1000 resampled datasets are shown at branch nodes.

Comparison of the nucleotide sequence of 16S rRNA gene of the strain KLDS1.0318 in National Centre for Biotechnology Information (NCBI) showed that it belonged to the genus *L. plantarum* (more than 99% similarity). The phylogenetic tree of KLDS1.0318 based on comparative analysis of the 16S rRNA gene (Figure 1) showed that the strain KLDS1.0318 and *L. plantarum* JCM 1149^T^ (D79210) belong to the same taxonomic group. The tree indicates that the strain KLDS1.0318 was assigned to the species *L. plantarum*.

### Levels of Cytokines in the Small Intestine

The levels of cytokines are represented in Table 2. Compared with NC group, CTX injection affected all the cytokines determined, causing significant (*p <* 0.05) reduction in IL-2, IL-4, IL-6, IL-10, TNF-α, and IFN-γ level. The levels of cytokines mentioned above except IL-6 were increased significantly (*p* < 0.05) in all of the KLDS1.0318 treated groups and PC group as compared to MC group. In addition, the bacterium of KLDS1.0318 ameliorated the decrease in the content of cytokines in a dose dependent manner. It is noted that 5 × 10^9^ CFU/mL of bacterium treatment (KLDS1.0318-H) achieved maximum amelioration against CTX-induced increase in the levels of cytokines in mice.

**Table 2:** Effect of *L. plantarum* KLDS1.0318 on levels of cytokines in small intestine in mice.

Cytokines	NC	MC	PC	KLDS 1.0318-L	KLDS 1.0318-M	KLDS 1.0318-H
IL-2 (pg/mL)	213 ± 11.2^c^	145 ± 14.1^a^	287 ± 12.2^d^	181 ± 11.2^b^	233 ± 11.6^c^	274 ± 17.3^d^
IL-4 (pg/mL)	161 ± 12.1^b^	134 ± 11.5^a^	209 ± 10.5^cd^	166 ± 11.3^b^	192 ± 10.4^c^	224 ± 19.1^d^
IL-6 (pg/mL)	301 ± 21.0^d^	177 ± 11.3^a^	267 ± 17.7^c^	193 ± 12.5^ab^	221 ± 14.7^b^	254 ± 15.3^c^
IL-10 (pg/mL)	386 ± 22.0^cd^	312 ± 18.1^a^	411 ± 12.7^d^	343 ± 13.4^b^	374 ± 17.9^c^	409 ± 18.4^d^
TNF-α (pg/mL)	319 ± 17.4^bc^	265 ± 17.7^a^	386 ± 18.7^d^	305 ± 17.8^b^	339 ± 15.5^c^	374 ± 20.0^d^
IFN-γ (ng/mL)	2.26 ± 0.21^d^	0.98 ± 0.06^a^	2.25 ± 0.23^d^	1.33 ± 0.11^b^	1.69 ± 0.13^c^	1.98 ± 0.15^d^


*NC, non-CTX + sterile saline; MC, CTX + sterile saline; PC, CTX + levamisole hydrochloride (40 mg/kg); KLDS1.0318-L, CTX + 5 × 10^*7*^ CFU/mL L. plantarum KLDS1.0318; KLDS1.0318-M, CTX + 5 × 10^*8*^ CFU/mL L. plantarum KLDS1.0318; KLDS1.0318-H, CTX + 5 × 10^*9*^ CFU/mL L. plantarum KLDS1.0318. Data are expressed as the mean ± SD (n = 10). Significant differences (p < 0.05) between the groups are indicated with different letters above the data.*

### Villus Height and Crypt Depth of the Small Intestine

**FIGURE 2:**
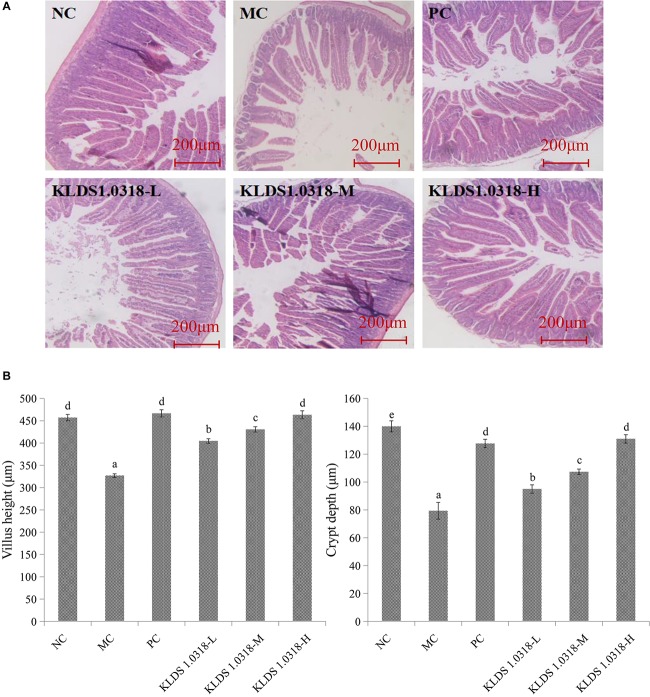
Effect of *L. plantarum* KLDS1.0318 treatments on villus height and crypt depth in the small intestine. **(A)** HE staining of jejunum sections, original magnification, 40×. **(B)** The villus height and the crypt depth. NC, non-CTX + sterile saline; MC, CTX + sterile saline; PC, CTX + levamisole hydrochloride (40 mg/kg); KLDS1.0318-L, CTX + 5 × 10^7^ CFU/mL *L. plantarum* KLDS1.0318; KLDS1.0318-M, CTX + 5 × 10^8^ CFU/mL *L. plantarum* KLDS1.0318; KLDS1.0318-H, CTX + 5 × 10^9^ CFU/mL *L. plantarum* KLDS1.0318. Data are expressed as the mean ± SD (*n* = 10). Significant differences (*p* < 0.05) between the groups are indicated with different letters above the bars.

Figure 2A shows the photomicrographs of HE-stained jejunum sections of mice. No microscopic lesions were observed in the jejunum of mice in NC group, which showed a normal histological morphology. The epithelium villi were high columnar, arranged neatly. While the mice in the MC group showed serious intestinal mucosa damage, obvious cell infiltration and edema relative to the NC group. The CTX plus low dose of bacterium group showed moderate injury in the small intestine. Notably, compared to NC group and the other four treatment groups, the villus height and the crypt depth significantly (*p* < 0.05) decreased in the MC group as shown in Figure 2B. Thus, these results showed that KLDS1.0318 can prevent intestinal injury partially. As the amount of bacterium increased in the oral administration, the amelioration effect of KLDS1.0318 becomes more apparent, especially with the high dose (5 × 10^9^ CFU/mL) KLDS1.0318-treated group.

### Number of Goblet Cells and Mucins Area of the Small Intestine

**FIGURE 3:**
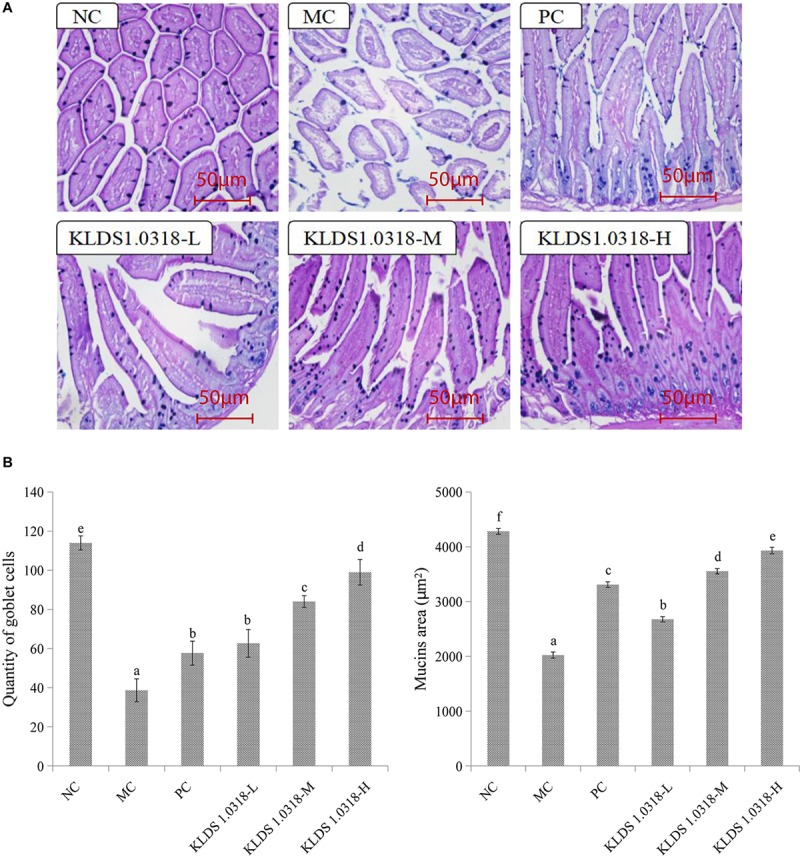
Effect of *L. plantarum* KLDS1.0318 treatments on the number of goblet cells and mucins area in the small intestine. **(A)** AB-PAS staining of jejunum sections, original magnification, 200×. **(B)** The number of goblet cells and the area of mucins. NC, non-CTX + sterile saline; MC, CTX + sterile saline; PC, CTX + levamisole hydrochloride (40 mg/kg); KLDS1.0318-L, CTX + 5 × 10^7^ CFU/mL *L. plantarum* KLDS1.0318; KLDS1.0318-M, CTX + 5 × 10^8^ CFU/mL *L. plantarum* KLDS1.0318; KLDS1.0318-H, CTX + 5 × 10^9^ CFU/mL *L. plantarum* KLDS1.0318. Data are expressed as the mean ± SD (*n* = 10). Significant differences (*p* < 0.05) between the groups are indicated with different letters above the bars.

The photomicrographs of AB-PAS-stained jejunum sections are represented in Figure 3A. Changes in morphology and structure of small intestine showed the same trend as the results obtained from HE staining. The MC group mice jejunum tissue structural integrity is impaired in comparison to the NC group. However, three groups of KLDS1.0318 treatment restored the intestinal damage with different degree, and significantly (*p* < 0.05) enhanced CTX-induced decrease in the number of goblet cells and mucins area in a dose dependent manner as displayed in Figure 3B. Hence, the results confirmed the preventive effects of KLDS1.0318 against CTX-induced intestinal injury.

### SCFAs Concentration

Table 3 shows the effect of different treatments on the levels of SCFAs in the mouse feces. A significantly (*p* < 0.05) reduction of fecal SCFAs (acetic acid, propionic acid, butyric acid and valeric acid) levels has been noted in the MC group as compared to NC group. On the other hand, SCFAs levels in all the KLDS1.0318 treatment groups and PC group were apparently higher than those in the PC group (*p* < 0.05).

**Table 3:** The concentration of acetic acid, propionic acid, butyric acid and valeric acid in the mouse feces of different groups.

SCFAs	NC	MC	PC	KLDS 1.0318-L	KLDS 1.0318-M	KLDS 1.0318-H
Acetic acid (μmol/g)	71.33 ± 3.41^b^	60.67 ± 2.49^a^	91.23 ± 3.77^c^	62.18 ± 4.50^a^	74.57 ± 4.66^b^	85.52 ± 5.11^c^
Propionic acid (μmol/g)	63.87 ± 2.58^b^	51.65 ± 2.67^a^	83.30 ± 3.31^d^	62.83 ± 3.25^b^	70.44 ± 5.10^c^	78.26 ± 4.41^d^
Butyric acid (μmol/g)	77.39 ± 3.16^d^	48.86 ± 2.41^a^	84.71 ± 4.10^e^	60.24 ± 3.56^b^	69.18 ± 3.73^c^	78.27 ± 4.12^d^
Valeric acid (μmol/g)	4.12 ± 0.15^bc^	3.43 ± 0.14^a^	4.71 ± 0.21^e^	3.94 ± 0.17^b^	4.30 ± 0.22^cd^	4.55 ± 0.23^de^


*NC, non-CTX + sterile saline; MC, CTX + sterile saline; PC, CTX + levamisole hydrochloride (40 mg/kg); KLDS1.0318-L, CTX + 5 × 10^*7*^ CFU/mL L. plantarum KLDS1.0318; KLDS1.0318-M, CTX + 5 × 10^*8*^ CFU/mL L. plantarum KLDS1.0318; KLDS1.0318-H, CTX + 5 × 10^*9*^ CFU/mL L. plantarum KLDS1.0318. Data are expressed as the mean ± SD (n = 10). Significant differences (p < 0.05) between the groups are indicated with different letters above the data.*

### Fecal pH

**FIGURE 4:**
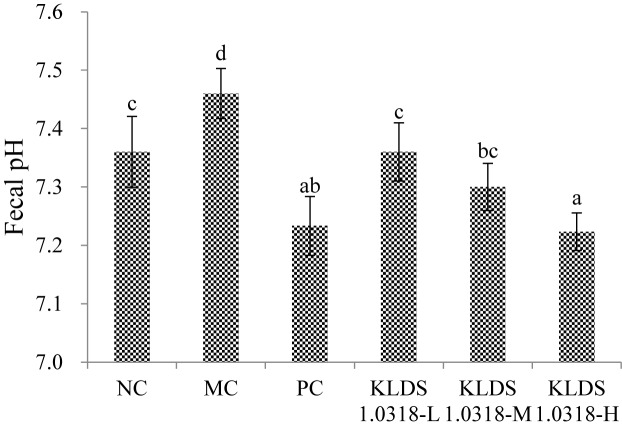
pH values in the mouse colon feces of different groups. NC, non-CTX + sterile saline; MC, CTX + sterile saline; PC, CTX + levamisole hydrochloride (40 mg/kg); KLDS1.0318-L, CTX + 5 × 10^7^ CFU/mL *L. plantarum* KLDS1.0318; KLDS1.0318-M, CTX + 5 × 10^8^ CFU/mL *L. plantarum* KLDS1.0318; KLDS1.0318-H, CTX + 5 × 10^9^ CFU/mL *L. plantarum* KLDS1.0318. Data are expressed as the mean ± SD (*n* = 10). Significant differences (*p* < 0.05) between the groups are indicated with different letters above the bars.

The effect of supplementation of KLDS1.0318 on mouse colonic pH is shown in Figure 4. It is observed that pH value was significantly (*p* < 0.05) increased in the MC group relative to the NC group. Compared with the MC group, however, the pH values were significantly (*p* < 0.05) decreased in all the three KLDS1.0318 treatment groups and PC group, especially in the groups treated with the high dose of KLDS1.0318.

### Ammonia Concentration

**FIGURE 5:**
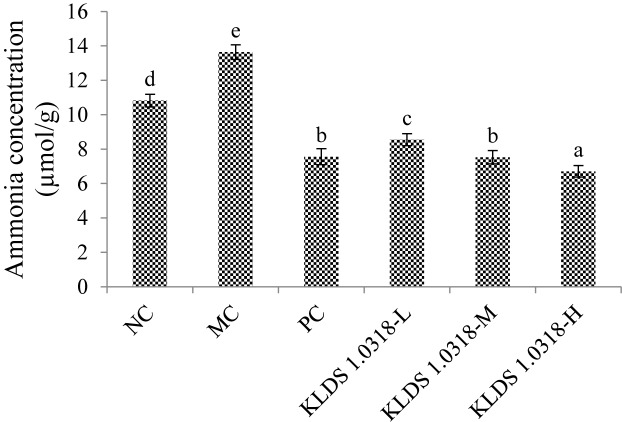
Cumulative production of ammonia in different groups. NC, non-CTX + sterile saline; MC, CTX + sterile saline; PC, CTX + levamisole hydrochloride (40 mg/kg); KLDS1.0318-L, CTX + 5 × 10^7^ CFU/mL *L. plantarum* KLDS1.0318; KLDS1.0318-M, CTX + 5 × 10^8^ CFU/mL *L. plantarum* KLDS1.0318; KLDS1.0318-H, CTX + 5 × 10^9^ CFU/mL *L. plantarum* KLDS1.0318. Data are expressed as the mean ± SD (*n* = 10). Significant differences (*p* < 0.05) between the groups are indicated with different letters above the bars.

The concentrations of ammonia in different groups are shown in Figure 5. It has been noted that CTX injection resulted in a significant (*p* < 0.05) increase of ammonia production in the MC group compared to the NC group. Nevertheless, the three different doses of bacterium reduced the ammonia production in a dose-dependent manner. It is clear that 5 × 10^9^ CFU/mL bacterium treatment effectively down-regulated CTX-induced increase in the ammonia concentration.

### Real-Time PCR Analysis

The abundance of bacteria communities in fecal samples is represented in Table 4. Compared to control and KLDS1.0318 group (5 × 10^9^ CFU/mL), the abundance of *Bifidobacterium* and *Lactobacillus* significantly (*p* < 0.05) decreased in CTX-treated group. Moreover, the abundance of *Escherichia* and *Enterococcus* significantly (*p* < 0.05) increased in CTX-treated group. However, the medium and high dose bacterium of KLDS1.0318 supplementation reversed CTX-induced changes in abundance of *Escherichia* and *Enterococcus* significantly (*p* < 0.05).

**Table 4:** Quantification of target bacteria from cecal contents in mice.

Genera [lg (copy no./ g feces)]	NC	MC	PC	KLDS 1.0318-L	KLDS 1.0318-M	KLDS 1.0318-H
*Bifidobacterium*	9.51 ± 0.27^c^	8.52 ± 0.17^a^	9.34 ± 0.25^c^	8.63 ± 0.22^a^	8.87 ± 0.19^ab^	9.19 ± 0.21^bc^
*Lactobacillus*	8.54 ± 0.22^c^	7.61 ± 0.32^a^	8.22 ± 0.28^d^	7.75 ± 0.37^ab^	7.92 ± 0.21^ab^	8.18 ± 0.23^bc^
*Escherichia*	9.45 ± 0.21^a^	10.72 ± 0.18^d^	9.55 ± 0.13^ab^	10.11 ± 0.24^c^	9.96 ± 0.19^bc^	9.74 ± 0.33^abc^
*Enterococcus*	7.11 ± 0.14^a^	7.92 ± 0.22^d^	6.91 ± 0.17^a^	7.63 ± 0.25^cd^	7.49 ± 0.18^bc^	7.22 ± 0.24^ab^


*NC, non-CTX + sterile saline; MC, CTX + sterile saline; PC, CTX + levamisole hydrochloride (40 mg/kg); KLDS1.0318-L, CTX + 5 × 10^*7*^ CFU/mL L. plantarum KLDS1.0318; KLDS1.0318-M, CTX + 5 × 10^*8*^ CFU/mL L. plantarum KLDS1.0318; KLDS1.0318-H, CTX + 5 × 10^*9*^ CFU/mL L. plantarum KLDS1.0318. Data are expressed as the mean ± SD (n = 10). Significant differences (p < 0.05) between the groups are indicated with different letters above the data.*

## Discussion

The gastrointestinal (GI) tract is an organ system which has important functional roles in the host. It is responsible for consuming and digesting foodstuffs, absorbing nutrients as well as expelling waste. The structural and immunological components in the GI tract are referred to as the “mucosal firewall” that consist of epithelial cells, mucus, antimicrobial peptides produced by epithelia, and immune cells, which play a significant role in limiting exposure to food-derived antigens, metabolites, and pathogens (Macpherson et al., 2009; Hooper and Macpherson, 2010). CTX is a commonly used chemotherapeutic agent for various forms of cancer. It targets effectively dividing neoplastic cells, but also affects the progenitor cell populations throughout the body (Decca et al., 2003). The intestinal epithelial cells are particularly susceptible hence chemotherapy can cause serious damage to the intestine, leading to the development of side effects such as nausea, vomiting and diarrhea (Logan et al., 2007; Triantafyllou et al., 2008). The ambition of this study was to determine the protective effects of *L. plantarum* KLDS1.0318 on the intestinal function in CTX-treated mice.

It is clear that type 1 helper T cells (Th1) and type 2 helper T cells (Th2) are two distinct subsets differentiated by activated CD4+ T cells, according to the differences in secretion of cytokines and immune functions: Th1 cells secrete the cytokines IL-2, IFN-γ, and TNF-α, which are mainly involved in cell-mediated immunity, while Th2 cells secrete the cytokines IL-4, IL-6, and IL-10, which are chiefly involved in humoral immunity (Kidd, 2003). The function of Th1/Th2 is generally at a kinetic equilibrium state to maintain the normal cellular and humoral immune responses in host. Some studies have demonstrated that *Lactobacillus strains* are capable of considerably influencing the Th1/Th2 immune responses (Borchers et al., 2002; Torii et al., 2007; Xie et al., 2015). This present study showed significant improvements in the levels of Th1 immune response cytokines IL-2, IFN-γ, and TNF-α in all of the *L. plantarum* KLDS1.0318-treated groups as compared to the MC group. Additionally, the secretion of Th2 immune response cytokines IL-4 and IL-10 were also significantly upregulated with three different treatments of *L. plantarum* KLDS1.0318 along with CTX exposure, while the level of IL-6 was significantly increased in the medium and high dose of KLDS1.0318 groups. The results indicated that *L. plantarum* KLDS1.0318 may maintain the normal intestinal immune function by stimulating the secretion of cytokines and regulating the Th1/Th2 balance.

It was observed in the current study that CTX extremely affected the growth as well as morphological integrity of the intestinal mucosa, which is consistent with a previous investigation (Zuo et al., 2014). The jejunum histological observations displayed abnormal morphological signs of cell infiltration and edema in the CTX-treated group, whereas the abnormal symptoms in the intestine partially disappeared with *L. plantarum* KLDS1.0318 treatment (increase villus height and crypt depth) in all the three groups. Our data indicated that oral administration of *L. plantarum* KLDS1.0318 significantly mitigated CTX-induced intestinal epithelia damage by increasing villus height and crypt depth. The results were in agreement with the earlier report that a probiotic strain of *L. plantarum* isolated from pickled vegetables could effectively reverse the villus height and crypt depth changes from treatment with CTX (Xie et al., 2016).

Goblet cells of mucosal surface are known to produce mucus which represents the primary shield not only limiting contact between the host and the commensal microbiota including bacteria, fungi, viruses, and other microbial but also preventing microbial translocation (Mcguckin et al., 2011). It has been demonstrated that *L. plantarum* NCU116 supplementation is capable of promoting the proliferation of goblet cells and the secretion of mucins proteins in the intestine of CTX-treated mice (Xie et al., 2016). In this study, compared to the MC group, a significant improvement in the number of goblet cells and the area of mucins was observed in all the probiotic-supplemented groups. The findings implicated that *L. plantarum* KLDS1.0318 could promote the repair of damaged intestinal mucosal induced by CTX.

SCFAs, a particularly versatile class of microbial metabolite, are derived from colonic microbial fermentation of dietary fibers and are likely to affect various aspects of host physiology broadly and significantly (Koh et al., 2016). SCFAs have been proven to limit GI inflammation by promoting the induction regulatory T cells and inhibiting the activation of macrophage and neutrophil (Arpaia et al., 2013; Smith et al., 2013). SCFAs can also directly act on certain pathogens via downregulating the expression of virulence genes (Gantois et al., 2006). A lack of SCFAs may enhance intestinal permeability and give rise to autoimmune type 1 diabetes (Cani et al., 2009). Furthermore, it has been previously reported that SCFAs could provide a host a number of benefits, acting as the metabolic fuel for different organs (muscle, kidney, heart, liver) and brain tissue, as well as offering bacteriostatic and bactericidal properties against certain microorganisms such as *Salmonella* and *E. coli* (Corrier et al., 1990). Some earlier studies showed that oral consumption of *L. plantarum* influenced the levels of fecal SCFAs by modulating the composition of the intestinal microbiota (increase the probiotic counts and decreased harmful bacteria) (Peng et al., 2016; Xie et al., 2016). It has been noted that different treatments of probiotic KLDS1.0318 markedly improved the production of SCFAs compared to the MC group in our study. Importantly, a significant decrease in fecal pH level might be the result of the production of SCFAs in the supplementation of KLDS1.0318 groups. The intestinal environment with a low pH value level is thought to be important for restraining the growth of some pathogens and affecting microbial enzymes’ activities (Hu et al., 2012). The supplementation with probiotic *L. casei* C1 and *L. plantarum* C4 strains can inhibit *Yersinia enterocolitica* via lowering pH level as a result of glucose fermentation has been reported (Bujalance et al., 2014). Our results showed that *L. plantarum* KLDS1.0318 increased the production of SCFAs and decreased the level of pH to maintain intestinal health.

Ammonia is a critical metabolite in the gut, which was reported to be toxic and potentially carcinogenic to intestinal epithelial cells (Visek, 1984). The increase of SCFA concentration raised the acidity in the gut, which promoted the use of ammonia by intestinal flora with ammonia as the nitrogen source, thus leading to a decrease in ammonia concentration (Brinkworth et al., 2009). It has been showed previously that administration with probiotics effectively reduced the production and absorption of ammonia to alter the gut flora of patients with hepatic encephalopathy (Sharma and Singh, 2016). Similarly, our data showed that supplementation of KLDS1.0318 significantly decreased the ammonia concentration in dose-dependent manner as compared to CTX alone-treated group. The result demonstrated that *L. plantarum* KLDS1.0318 intervention could be beneficial for intestinal epithelia by inhibiting the production of ammonia.

Probiotics may function through competitive exclusion of pathogens, either through direct inhibitory or competitive activity exerted by probiotic strains, or through the influence of probiotics on the endogenous commensal microflora, or by changing metabolite production by the intestinal microbiota (Sarah et al., 2008; Peng et al., 2016). Our findings of CTX significantly affected the intestinal microbiota composition, which is in consistent with an earlier study (Yang et al., 2013). Additionally, this study found that supplementation with *L. plantarum* KLDS1.0318 increased the abundance of *Bifidobacterium* and *Lactobacillus* and decreased the abundance of *Escherichia* and *Enterococcus* in cecal content. Similarly, it has been stated earlier that the supplementation of *L. plantarum* reduced fecal potentially pathogenic bacteria (*Enterobacteriaceae*) and increased fecal probiotic counts (Thanh et al., 2009; Xie et al., 2016).

## Conclusion

In conclusion, the present study showed that the oral administration of *L. plantarum* KLDS1.0318 normalized the parameters altered by CTX-induced toxicities, strengthening intestinal health by regulating the Th1/Th2 balance, ameliorating the intestinal morphology and improving profiles of intestinal microbiota and metabolism. Therefore, our findings suggested that the administration of *L. plantarum* KLDS1.0318 could be of significant advantage in reducing intestinal immunity impairment caused by cyclophosphamide.

## Ethics Statement

This study was carried out according to the Animal Care Review Committee, Northeast Agricultural University.

## Author Contributions

CL supervised the whole experiments. YM contributed to paper writing. YM, JW, ZW, and GZ performed the practical work and completed the experiments. LL and GH provided help during the experiments. CL provided place, lab facilities, and funding for the present work.

## Conflict of Interest Statement

The authors declare that the research was conducted in the absence of any commercial or financial relationships that could be construed as a potential conflict of interest.
